# Bactericidal effect of low-temperature atmospheric plasma against the *Shigella flexneri*

**DOI:** 10.1186/s12938-023-01185-w

**Published:** 2023-12-09

**Authors:** Yan Chen, Yuanyuan He, Tao Jin, Chenwei Dai, Qinghua Xu, Zhengwei Wu

**Affiliations:** 1 Joint Laboratory of Plasma Application Technology, Institute of Advanced Technology, University of Science and Technology of China, Hefei, China; 2 School of Nuclear Science and Technology, University of Science and Technology of China, Hefei, China; 3 Department of Geriatrics, The First Affiliated Hospital of USTC, Division of Life Sciences and Medicine, University of Science and Technology of China, Hefei, China; 4 Anhui Academy of Medical Sciences, Hefei, China

**Keywords:** Cold atmospheric plasma, Dielectric barrier discharge, *Shigella flexneri*, Mortality rate, Reactive oxygen species

## Abstract

**Background:**

*Shigella flexneri* (*S. flexneri*) is a common intestinal pathogenic bacteria that mainly causes bacillary dysentery, especially in low socioeconomic countries. This study aimed to apply cold atmospheric plasma (CAP) on *S. flexneri* directly to achieve rapid, efficient and environmentally friendly sterilization.

**Methods:**

The operating parameters of the equipment were determined by plasma diagnostics. The plate count and transmission electron microscope were employed to calculate bacterial mortality rates and observe the morphological damage of bacterial cells. Measurement of intracellular reactive oxygen species (ROS) and superoxide anions were detected by 2,7-dichlorodihydrofluorescein (DCFH) and Dihydroethidium fluorescence probes, respectively. The fluorescence intensity (a. u.) reflects the relative contents. Additionally, the experiment about the single effect of temperature, ultraviolet (UV), and ROS on bacteria was conducted.

**Results:**

The peak discharge voltage and current during plasma operation were 3.92kV and 66mA. After discharge, the bacterial mortality rate of 10, 20, 30 and 40 s of plasma treatment was 60.71%, 74.02%, 88.11% and 98.76%, respectively. It was shown that the intracellular ROS content was proportional to the plasma treatment time and ROS was the major contributor to bacterial death.

**Conclusion:**

In summary, our results illustrated that the plasma treatment could inactivate *S. flexneri* efficiently, and the ROS produced by plasma is the leading cause of bacterial mortality. This highly efficient sterilization method renders plasma a highly promising solution for hospitals, clinics, and daily life.

## Background

*Shigella*, a facultative anaerobic gram-negative bacterium with highly pathogenic and persistent infection, accounts for diarrheal diseases and food safety today. In 2016, *Shigella* cases resulted in 212 438 deaths among all ages and 60 000 deaths in children under 5 years old [1]. According to the World Health Organization (WHO), each year shigellosis causes approximately 700 000 deaths worldwide [2]. *Shigella* was usually classified into four species: *Shigella flexneri*, *Shigella sonnei*, *Shigella boydii*, and *Shigella dysenteriae*, and the first two were most common in Asia [3]. In developing and developed countries, 60% and 16% of the cases of diarrheal diseases were caused by *S. flexneri*, respectively [4]. *S. flexneri* is commonly found in vegetables, fruits and other easily attachable equipment. It can survive for up to 2 weeks in vegetables, fruits, and pickles, and can multiply. With the increasing global issue of bacterial resistance, utilizing drugs to hinder *S. flexneri* as a solution for eradicating bacteria might no longer be efficacious. Although antibacterial treatment could reduce the risk of serious clinical complications and death, multi-antibiotic-resistant bacterial pathogens have inevitably developed resistance to every new drug introduced in the clinic due to the widespread use of antibiotics [5]. Moreover, the research and development of new drugs takes decades and costs a lot, so finding new strategies independent of bacterial resistance to antibiotics to alleviate the situation is imperative. Cold atmospheric plasma (CAP) has been found to have this potential due to its powerful bactericidal action [6]. It can sterilize human tissue with minimal or no damage and eliminate bacteria or spores in a broad range of food materials without altering the primary physical–chemical properties of the food and the nature of the equipment. Bacteria inactivation efficiency of direct atmospheric pressure plasma treatment is extremely strong [7].

Plasma, the fourth state of matter, contains ions, electronics, ultraviolet, and many active substances such as free radicals. Dielectric barrier discharge (DBD), generated through the introduction of an insulating material in the space between two electrodes, represents a common form of gas discharge producing room temperature plasma, and it can be performed under diverse gas pressure ranges. The insulating dielectric may envelop the surface of one electrode or be suspended between the two electrodes and can be composed of rubbers, glass, quartz or ceramics. Currently, it has found widespread application in areas such as surface treatment, ozone generation, exhaust gas purification, and ultraviolet light sources [8, 9].

CAP has been widely applied in wound healing, adjuvant therapy, and purification of hospital environments due to its procoagulant function, apoptosis-inducing ability, and sterilization [10–12]. In terms of sterilization, plasma has shown sound therapeutic effects on keratitis, skin infections, upper respiratory tract infections, and implant infections, mainly because of its effectiveness against pathogenic bacteria (such as *Staphylococcus aureus*, *Pseudomonas aeruginosa*, and *Candida albicans* that cause these diseases, etc.) has excellent bactericidal activity without toxic side effects on tissues and organs [13–17]. It has been reported that the mechanism of plasma sterilization is mainly focused on the oxidation of microbial cells by active particles of plasma [18, 19]. Compared with the traditional sterilization methods such as autoclave sterilization or radiation sterilization, sterilization by plasma has the advantages of short time and high efficiency, close to room temperature, thus without thermal damage, and no harm to the surrounding normal cells, as well as the by-products which will not affect the environment [14, 20, 21]. Therefore, it is worthwhile to study the treatment of microorganisms with plasma technology, which has enormous potential in assisting the antibiotic inactivation of pathogenic organisms.

Currently, plasma works on bacteria mainly by indirect treatment, treating bacterial solution, or using plasma-activated solutions as the bacterial solvent. Direct treatment of bacteria is relatively rare, but it has also been proved that this method can kill the bacteria efficiently [22]. Hence, the plasma can be applied in the liquid phase environment and directly used for sterilization in the non-liquid phase environment, such as equipment sterilization, space sterilization, etc. No matter which treatment method, the inactivation of bacteria is closely related to the active substances produced by plasma, including the first-generation produced during discharge and the second-generation reactive oxygen species (ROS) grown by chemical reaction, and they could induce oxidative stress in bacteria which oxidize cellular lipids and proteins [23]. It interferes with the balance of the regular redox system in cells and stimulates the production of ROS in cells. When intracellular ROS accumulates to a certain amount, it will cause DNA damage, inhibit gene expression, lead to protein misfolding, and even affect protein synthesis, causing severe damage to the cell structure and leading to bacterial death [24].

In this study, we explored different plasma treatment times to observe the extent of damage to bacteria. Then, observation of cell morphology and measurement of intracellular active substance content demonstrated alterations in cells after plasma treatment. Determination of the active ingredient contents in the cell was done to study the composition of the substances responsible for the bactericidal effect of the plasma. Furthermore, experiments on the impact of plasma-generated temperature, UV, and ROS on bacteria alone were conducted to determine which was responsible for the bacterial death. In conclusion, the study demonstrated that CAP can rapidly and efficiently inactivate *S. flexneri* in a non-liquid phase environment and elucidated the mechanism of action that results in the inactivation of the bacteria, which is critical for sterilization and disinfection of CAP in both routine and clinical settings.

## Results

### CAP properties analysis

**Fig. 1:**
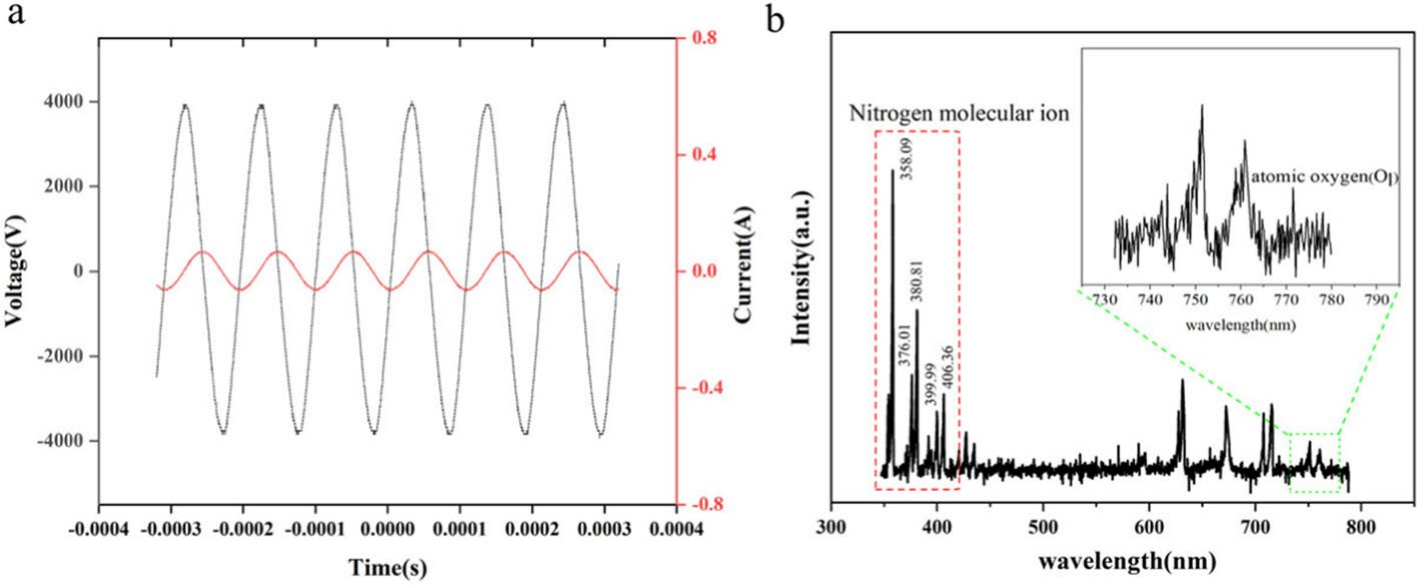
**a** Current and voltage waveform of CAP. **b** Emission spectrum of CAP

Connecting an oscilloscope and turning on the power, the peak excitation voltage and discharge current of the device were detected as 3.92 kV and 66 mA, respectively (Fig. 1a). Excitation voltage refers to the electric potential applied to a gas to initiate the process of ionization, the required excitation voltage depends on the type of gas, its pressure, and the configuration of the electrodes used to apply the voltage. At the same time, discharge current is the flow of electric charge through the plasma once formed. It measures how many electrons and ions are moving through the plasma. The magnitude of the discharge current depends on the excitation voltage and the properties of the plasma, such as its conductivity. Optical emission spectroscopy (OES) was generally used to identify the composition of reactive species generated by plasma discharge, and the optical spectra was recorded for emission from the DBD in the wavelength range from 300 to 800 nm in this study (Fig. 1b).

### Bacterial inactivation rate

**Fig. 2:**
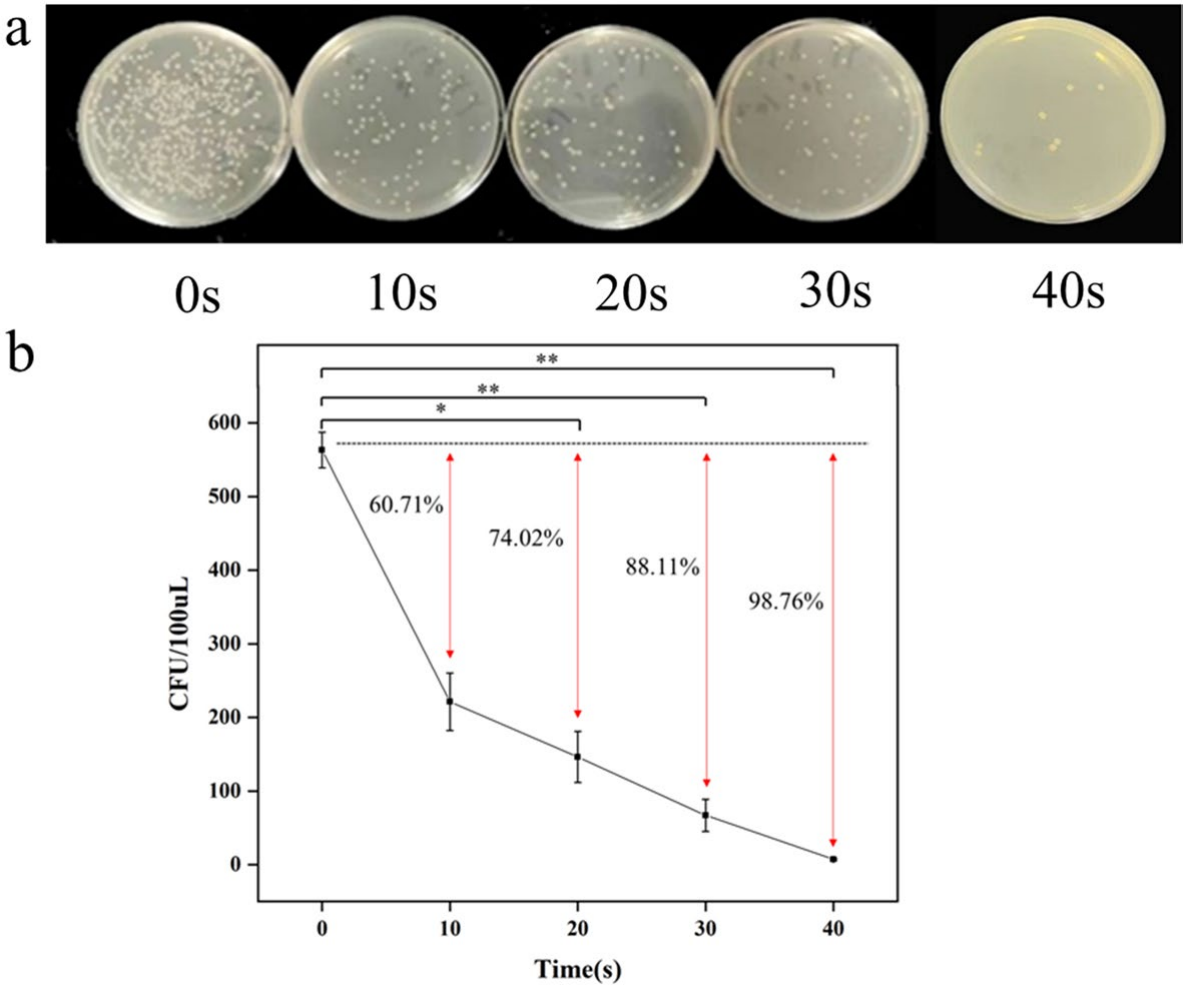
**a** Colonies of *S. flexneri* on the NA plate after plasma treatment with 0, 10, 20, 30, 40 s. **b** Colony counts after plasma treatment with different time and effects of CAP on the inactivation rate of bacteria in non-liquid environment. Data were represented by the average and standard deviation (*n* = 3)

Apparently, the bacterial mortality rate gradually increased with plasma treatment time (Fig. 2a). After treatment for 10, 20, 30, and 40 s, the concentration of bacteria decreased from 563 colonies to 193, 146, 67, and 7 on average. To further explore the bactericidal performance of plasma, we calculated the bacterial inactivation rate of plasma treatment (Fig. 2b). After treatment for 10 s, the mortality rate of bacteria was 60.71%, suggesting that the survival rate was less than half. When the treatment time reached 20 s, the mortality rate was 74.02%, and that of 30 s was 88.11%, indicating that the survival rate was as low as 10%. The sterilization rate can reach more than 98% after 40 s of treatment, and almost all bacteria were inactivated.

### TEM images of bacteria

**Fig. 3:**
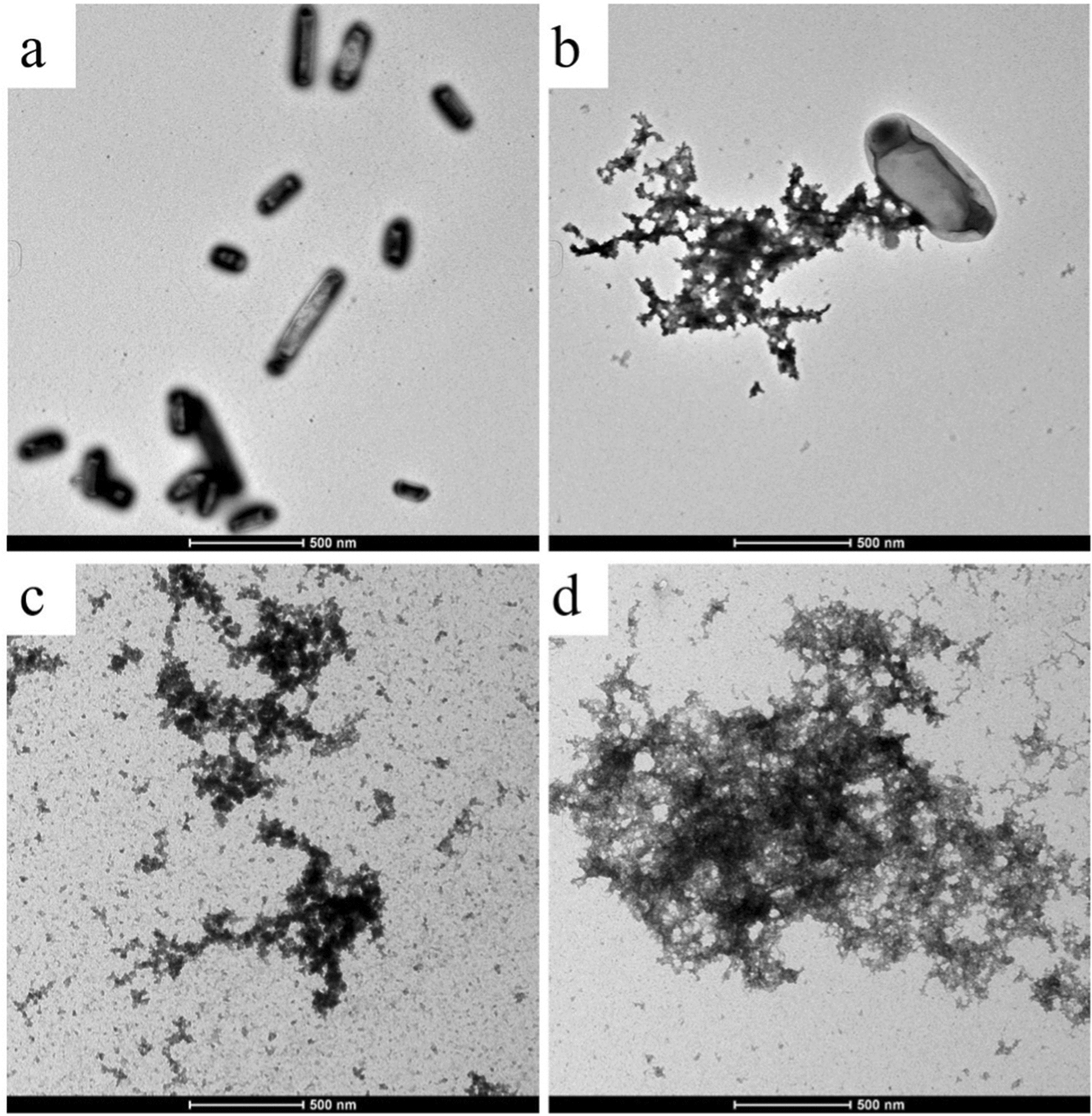
TEM images of bacterial cell structure. **a** Bacterial cell structure of untreated. **b** Bacterial cell structure treated by CAP for 10 s **c** Bacterial cell structure treated by CAP for 20 s **d** Bacterial cell structure treated by CAP for 30 s

After treatment, cell morphology was seriously damaged compared with the untreated group. It can be seen that the cells in the untreated group had a typical rod shape. After 10 s of treatment (Fig. 3a), some cells showed signs of damage (Fig. 3b), while after 20 and 30 s of treatment (Fig. 3c, d), cells exhibited significant structural damage, with some appearing darker in colour, indicating a lack of structurally intact cells.

### Reactive species in bacterial cells

**Fig. 4:**
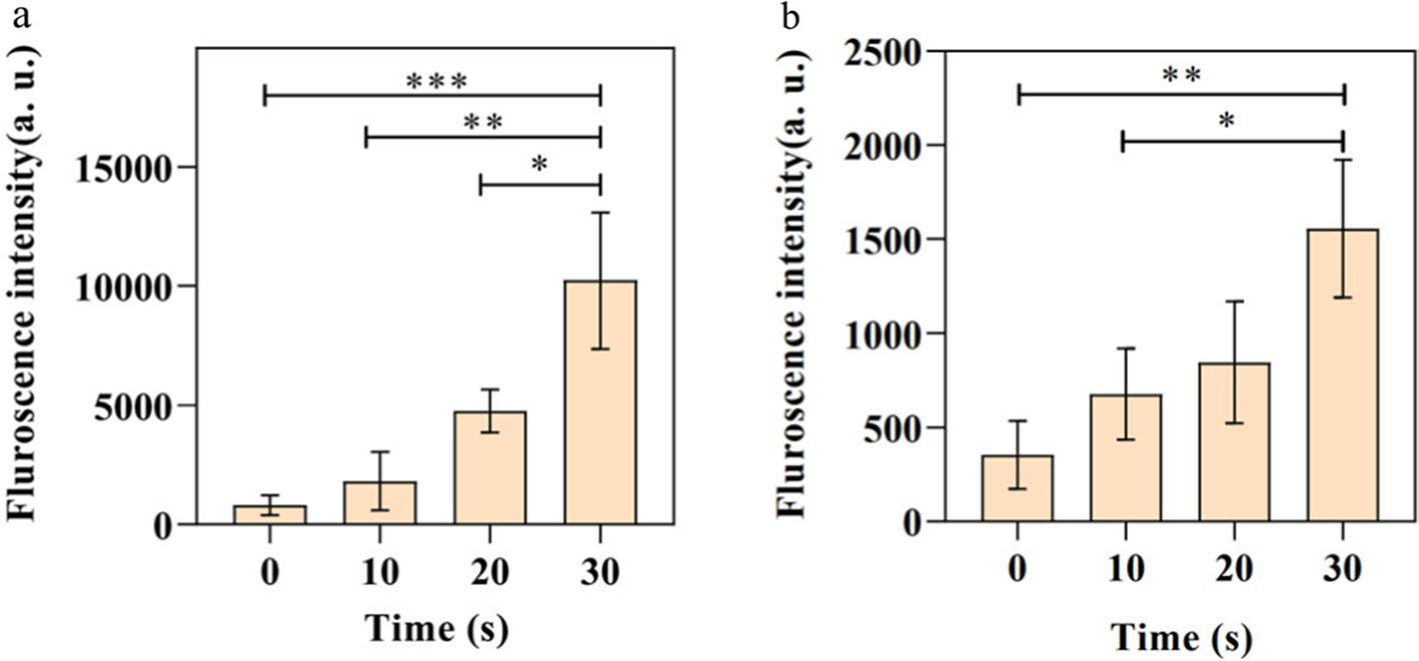
The intracellular content of **a** ROS and **b** superoxide anion O_2_^−^ after plasma treatment with different time. Data were represented by the average and standard deviation (*n* = 3)

As illustrated, after plasma treatment, the intracellular contents of ROS (Fig. 4a) and superoxide anion (Fig. 4b) increased compared with the untreated group, and the content increase was proportional to the action time. Within 30 s of treatment, the fluorescence intensity of ROS and superoxide anion was nearly 13-fold and fourfold higher than that of the untreated group. Excessive levels of reactive oxygen species can lead to cellular damage.

### The effect of temperature, UV, and ROS on the bacteria

**Fig. 5:**
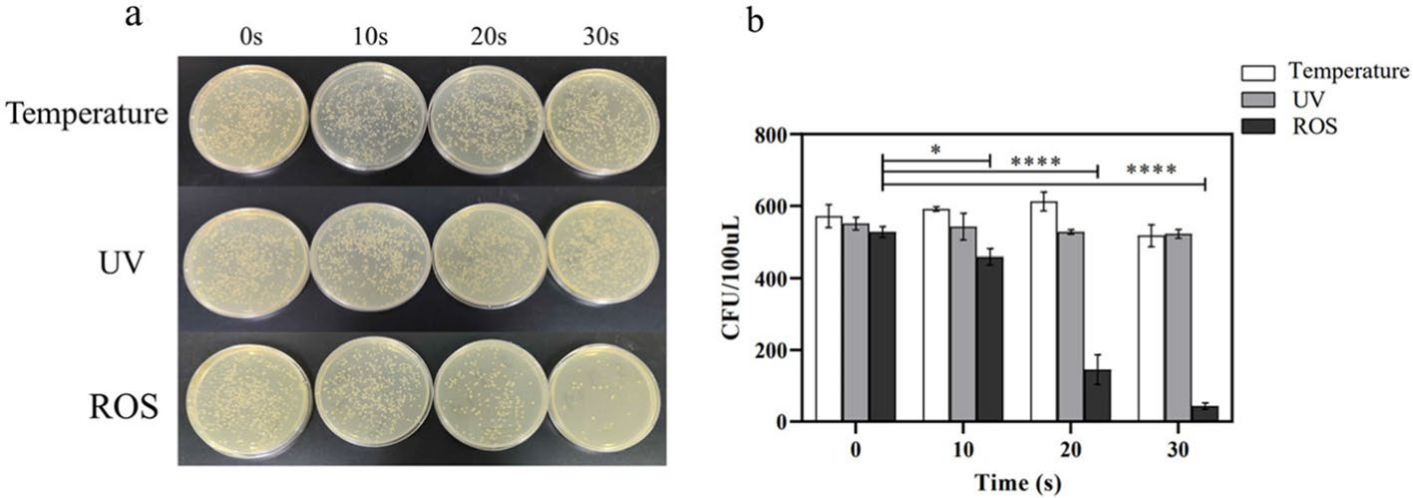
**a** Colonies of *S. flexneri* on the NA plate after plasma treatment with the effect of temperature, UV, and ROS of the DBD. **b** Colony counts after treatment under different conditions. Data were represented by the average and standard deviation (*n* = 3)

The surface temperature of the DBD device was measured after 10, 20, 30 s of operation. And the recorded temperatures were 24.7 °C, 30.8 °C, and 32.6 °C, respectively. Other factors aside, bacterial death was not affected by the temperature generated by the device (Fig. 5a). Similarly, the results showed that the effect of UV on bacterial mortality was not huge, while ROS was the leading cause of bacterial mortality (Fig. 5b). The bacterial mortality rates of 20 s and 30 s treatment were 72.49% and 91.84%, respectively, which were consistent with the previous results.

## Discussion

During the discharge process, the waveforms of the current and voltage did not fluctuate greatly, indicating that plasma was relatively stable in production. In the OES spectra of the discharge plasma, the strong emission of the second positive band N_2_, mainly composed of nitrogen molecules, was displayed at 340–410 nm. [25]. The second positive band N_2_ is a well-known series of bands in nitrogen whose leptonic spectrum corresponds to the transition from the *C*^3^П_*μ*_ electronic state to the *B*^3^П_*g*_ electronic state. This series of bands is observed in the ultraviolet and visible regions of the spectrum and is commonly studied in plasma physics and spectroscopy due to its prominence in nitrogen discharges. Moreover, the characteristic emission peak of atomic oxygen was exhibited at 777 nm [26]. This illustrated the complexity of the plasma composition, which contained a variety of active substances.

It can be seen that the inactivation effect of CAP on *S. flexneri* was powerful, and the killing ability increased with the prolongation of the working time. To achieve identical sterilization results, autoclaving and antibiotic antibacterial methods had limitations. High-pressure sterilization took approximately two hours, while complete sterilization was challenging to accomplish with antibiotics due to the emergence of drug resistance, and antibiotics were difficult to decompose, causing environmental pollution with excessive use. Therefore, CAP can be applied directly in the sterilization of produce, equipment and other items, without compromising the quality of food or materials. Regarding the treatment of bacterial infections in vivo in a clinical setting, further experiments are required. First and foremost, it is essential to deliver the CAP to the target site accurately. The plasma operating parameters must also be regulated to ascertain a safe dosage that does not harm normal tissues [27].

The findings from the transmission electron microscopy indicated the rupture of bacterial cells following plasma treatment. It was not difficult to explain the result because *Shigella* was a gram-negative bacterium with a thin cell wall, and plasma could oxidize the cell membrane's lipid and damage its integrity [28]. The longer the working time of CAP acted, the more severe the cell inactivation was, and it also illustrated that CAP was efficient in inactivating *S. flexneri*.

The active species plasma generated can be classified as long-lived and short-lived species. The former includes H_2_O_2_, NO_2_^−^, NO_3_^−^, and the latter contains ·OH, O_l_, ONOO.^−^, etc. ROS was a very representative and important indicator for detecting active species, so this study detected the content of ROS and superoxide anion. The result showed that the intracellular content of ROS after plasma treatment markedly surpassed that of the control group. Plasma-generated reactive species could damage cells through oxidizing lipids and proteins on the cell membrane surface. Still, it can also induce the accumulation of active substances in cells, which play an important role in mediating cell death [29]. ROS is produced through redox reactions in the inflammatory process, cell ischemia processes, and various crucial developmental processes, such as cell differentiation and cell signal pathway [30]. Low levels of ROS can promote cell proliferation and tissue repair, while high levels will cause cell death and apoptosis [31, 32].

Overall, in combination with the fragmented cellular morphology observed by electron microscopy and the detected increase in intracellular ROS content, it is hypothesized that plasma-generated ROS cause cell death by oxidizing cell membrane proteins and disrupting the balance of oxidative reactions within the cells.

Theoretically, the heat and UV contained in CAP affect the survival of the bacteria, but the experiment demonstrated that the ROS in the plasma was the main component that inactivated *S. flexneri*. *Shigella* was found to be stable over a wide range of temperatures (4–50 °C) and pH values (pH 3–11) [33]. Therefore, the effect of temperature generated by DBD on the survival of bacteria was insignificant, which confirms the literature that the survival of *Shigella* was not affected in an environment below 37 °C [34]. The UV emitted by the device had a negligible impact on bacterial mortality rate, whereas ROS exerted a significant effect on bacterial mortality. Combined with the measured intracellular ROS levels after plasma treatment, it can be assumed that the plasma-generated ROS induced an increase in intracellular ROS and caused bacterial death.

Currently, the study primarily investigates the effect of low temperature plasma on the bacterial solution via direct and indirect treatment. Indirect treatment mainly involves the application of plasma activated water (PAW) to bacterial cells. It has demonstrated that plasma can effectively eliminate common pathogenic bacteria in liquid phase environment, including *Staphylococcus aureus* and *Pseudomonas aeruginosa*. The inactivation efficacy of plasma relies on system and process variables, such as gas composition, power input, exposure time, and the intrinsic characteristics of the targeted microbial cells, precisely the delivery dose and cell type [35]. Although plasma-generated ROS can lead to oxidative stress and the death of bacterial cells, it has been reported that some bacteria can also produce anti-oxidative stress responses and finally exhibit tolerance to CAP [36]. Yang et al. discovered that bacteria expressing Staphyloxanthin or its yellow pigment intermediates demonstrated resistance to CAP [37]. Additionally, the survival of bacteria largely depends on the modification of their phenotype. In high oxygen concentration environments created by CAP, bacteria undergo changes in energy allocation [38]. A significant amount of energy is allocated to antioxidant activity, indicating a large up-regulation of antioxidant genes in gene expression. The alteration in the balance of intracellular energy allocation diminishes the energy accessible for bacterial metabolism, which is believed to result in a viable but non-culturable (VBNC) state [39]. Therefore, although our experimental findings demonstrated that *S. flexneri* displays greater sensitivity to CAP, further testing can be carried out by culturing the treated bacteria to observe any resultant alterations.

## Conclusion

In summary, this study demonstrated that CAP treatment can significantly inactivate *S. flexneri* in a non-liquid environment. Moreover, the bactericidal ability is powerful, which means that only a small dose is needed to achieve the daily sterilization of equipment and environment. Based on the cell morphology observed by electron microscopy and the results of intracellular ROS content measurement, it was assumed that the elevated ROS caused the intracellular redox imbalance of the bacteria, and the cell membrane structure was damaged, thus leading to the death of the bacteria. The findings of the single inhibition experiments of temperature, UV and ROS, which are the three main components of the plasma, also proved that the temperature and UV have no effect on bacterial inactivation, and ROS is the main contributor to bacterial mortality. Therefore, as a non-traditional sterilization method, plasma has the advantages of low cost, minor side effects, and short treatment time, indicating its broad potential for various environments. However, more specific mechanisms of plasma on bacterial cells have yet to be revealed, which will be the next point of our work.

## Methods

### Plasma device

**Fig. 6:**
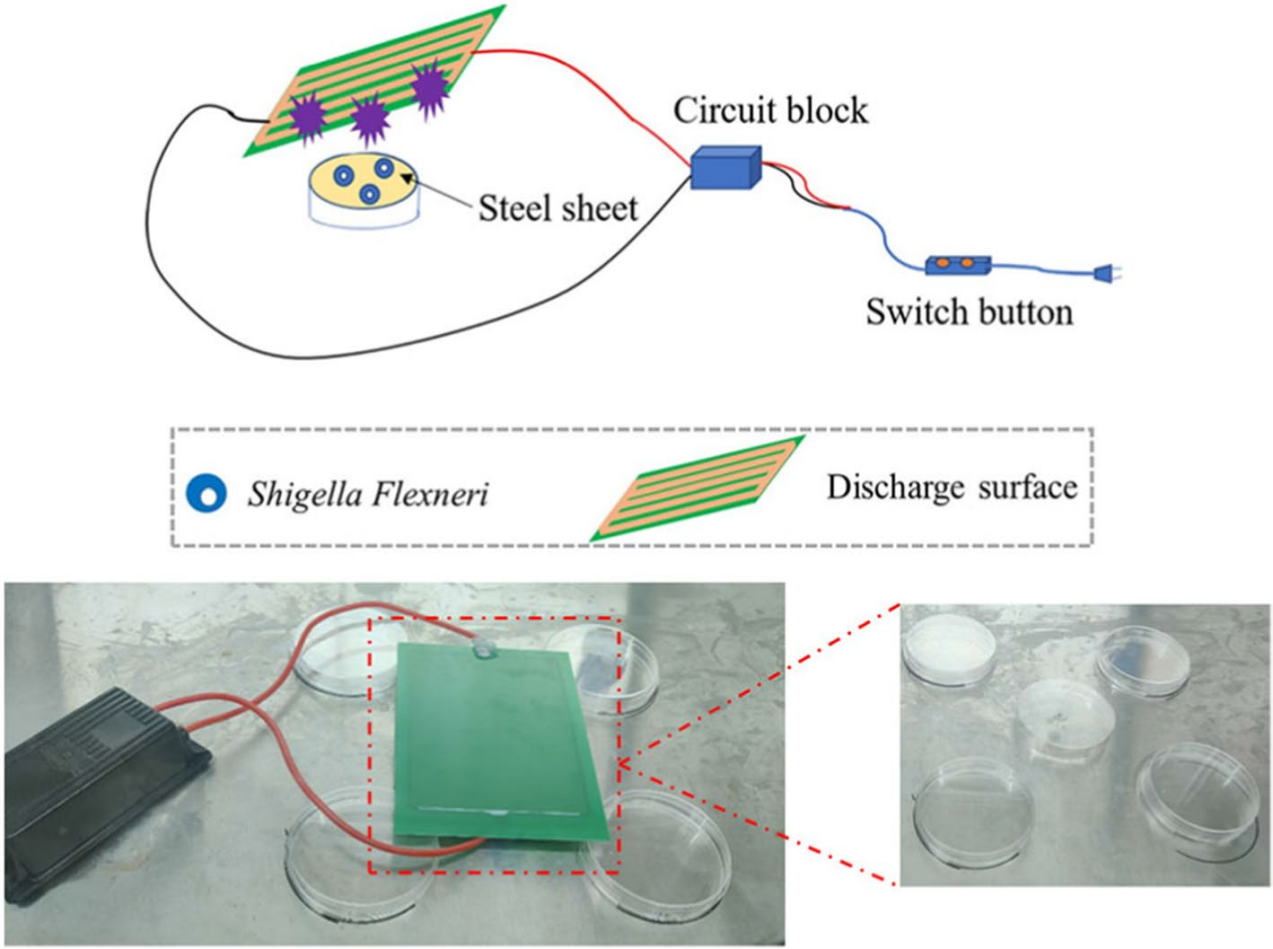
Image of plasma device and treatment to the *S. flexneri*

The CAP device was designed with a board containing high- and low-voltage sides, both made of copper (Fig. 6). The outside of the high-voltage side was a piece of copper sealed with epoxy resin, while the surface of the low voltage side consisted of many copper strips evenly on it, and the plasma was generated from it when the power was turned on.

The device operated through dielectric barrier discharge (DBD), and the air was the only reactant. The oscilloscope was applied to measure the current and voltage of the device. The device was fixed 5 cm away from the lens (radius 2.5 cm), and the lens was aligned with the center of the DBD discharge device. In the dark environment, the blue and purple light emitted from the area of the DBD discharge surface covered by the lens range was focused by the lens, and the emission spectrum in the spectral range of 320–1020 nm was measured by a charge-coupled device (CCD) detector (spec10:1000b; Princeton instrument) 27 cm away from the lens.

### Bacterial preparation and treatment

The microbial strain used in this study was *Shigella flexneri*. A small amount of bacterial broth stored at −20 ℃ was taken to spread on the Nutrition Agar (NA) plate and incubated at 37 °C for 18–24 h. The surface of the medium was rinsed with sterile phosphate-buffered solutions (PBS), and the concentration of bacteria was adjusted to 1.0McF (3 × 10^8^ CFU/mL) using a turbidimeter. After 100-fold gradient dilution, 10 µL was placed on a sterilized round steel sheet (Φ12mm) and air-dried naturally. The steel sheet was placed on a sterile petri dish with DBD equipment about 2 mm from the surface and treated for 10, 20, 30, and 40 s.

### Detection of inactivation rate

After treatment, the steel sheet was put into a 10 mL centrifuge tube containing 1 mL mixed solution of PBS and 0.1% Tween-80. The bacteria were rinsed to the answer from the surface of the steel sheet by vortex shocking. Each group was serially diluted, and 100 µL of each dilution was spread on the NA plate. All samples were incubated overnight at 37 °C, and colony-forming units (CFU) were counted on the plates [40]. The inactivation rate (%) of bacteria was calculated by the formula: 1-CFU (treated group)/CFU (untreated group) × 100%. As nearly all of the bacteria perished within 40 s treatment and were challenging to collect, the subsequent assay treatment groups featured only 10, 20 and 30 s.

### Transmission electron microscope (TEM)

The bacterial solution of the three experimental groups after plasma treatment and the untreated control group were collected and centrifuged at 8000 G for 10 min, the supernatant and 5 µL PBS were added to make the suspension. The sample suspension was sucked and dropped onto the copper screen, a few minutes later, the excess liquid was absorbed by filter paper, and the negative dye solution was dropped to dye for 2 min. After drinking the excess dye solution, it was used for electron microscope observation after drying.

### Determination of intracellular reactive species

Due to the treatment method of this experiment was to operate the bacteria in a non-liquid environment, only the content change of the active substances produced by plasma work entering the bacterial cells was measured. The Reactive Oxygen Species assay kit (Beyotime Biotechnology, Biotechnology, Shanghai, China) measured intracellular ROS content [41]. The fluorescent probe DCFH-DA was diluted with PBS to a final concentration of 10 µM. Bacteria cells were suspended in the diluted probe solution with a concentration of 10^6^ cells/mL and were incubated at 37 °C for 30 min. As described above, the steel sheets of the treatment group for each period were rinsed with different volumes of mixed solution of PBS and 0.1% Tween-80 for each group to contain the same number of living cells. The fluorescence intensities were detected using a microplate reader at the excitation wavelength of 488 nm and the emission wavelength of 525 nm. Set a hole, adding an equal volume of PBS solution as the blank when reading. A Dihydroethidium (Beyotime Biotechnology, Biotechnology, Shanghai, China) assay kit was applied for superoxide anion O_2_^−^ determination. Cells were loaded with the DHE probe containing 5 µM and incubated at 37 °C for 30 min for reading. The excitation/emission wavelengths were 535/610 nm.

### Determination of the antibacterial effect of temperature, UV, ROS

To analyze the natural impact of the temperature, UV, and ROS generated by the DBD, we experimented by adding additional barriers to allow a single ingredient to act on bacteria. After adding drops of the bacterial solution to the steel sheet placed on a petri dish, we detected the effect of UV, ROS, and temperature alone on the bacteria by adding a custom-made quartz glass lid to isolate ROS, a layer of less dense paper to isolate UV and a plastic cover to isolate UV and ROS. After treatment, the bacteria were collected and prepared according to the above description. They were diluted 100-fold and incubated in plates overnight. In addition, the surface temperature of DBD after 10, 20, and 30 s of plasma operation was measured using an infrared thermometer.

### Statistical analysis

All measurements for this study were conducted in triplicate. The experimental data were calculated and recorded as mean ± standard deviation (SD) using Origin Pro 2018. All treatments were statistically analyzed by GraphPad Prism 8.0, and an analysis of variance (ANOVA) was conducted to study differences with different periods, and the difference was expressed as *p < 0.05, **p < 0.01, ***p < 0.001, ****p < 0.0001.

## Data Availability

The data used and/or analyzed during the current study are available from the corresponding author upon reasonable request.

## Abbreviations

CAP: Cold atmospheric plasma
CCD: Charge-coupled device
DBD: Dielectric barrier discharge
ROS: Reactive oxygen species
